# Low-Calorie Beverage Consumption, Diet Quality and Cardiometabolic Risk Factors in British Adults

**DOI:** 10.3390/nu10091261

**Published:** 2018-09-07

**Authors:** Linia Patel, Gianfranco Alicandro, Carlo La Vecchia

**Affiliations:** Department of Clinical Sciences and Community Health, Università degli Studi di Milano, 20133 Milan, Italy; gianfranco.alicandro@unimi.it (G.A.); carlo.lavecchia@unimi.it (C.L.V.)

**Keywords:** low-calorie beverages, diet quality, cardiometabolic markers

## Abstract

Low-calorie beverages (LCBs) are promoted as healthy alternatives to sugar-sweetened beverages (SSBs); however, their effects on diet quality and cardiometabolic profile are debatable. This study aimed to verify the association between LCB consumption, diet quality and cardiometabolic risk factors in British adults. Data analysis from 5521 subjects aged 16 and older who participated in two waves of the National Diet and Nutrition Survey Rolling Programme (2008–2012 and 2013–2014) was carried out. Compared with SSB consumption, LCB consumption was associated with lower energy (mean difference: −173 kcal, 95% confidence interval, CI: −212; −133) and free sugar intake (−5.6% of energy intake, 95% CI: −6.1; −5.1), while intake of other nutrients was not significantly different across groups. The % difference in sugar intake was more pronounced among the young (16–24 years) (−7.3 of energy intake, 95% CI: −8.6; −5.9). The odds of not exceeding the UK-recommended free sugar intake were remarkably higher in the LCB as compared to the SSB group (OR: 9.4, 95% CI: 6.5–13.6). No significant differences were observed in plasma glucose, total cholesterol, LDL, HDL or triglycerides. Our findings suggest that LCBs are associated with lower free sugar intake without affecting the intake of other macronutrients or negatively impacting cardiometabolic risk factors.

## 1. Introduction

Nearly two-thirds of adults in the United Kingdom are either overweight or obese [1]. Obesity is an independent risk factor for many health problems including cardiovascular disease, type 2 diabetes and certain cancers [2].

Free or added sugars have been acknowledged as a readily available source of energy, which accounts for a large percentage of daily energy intake, leading to excess calories, weight gain and obesity [3]. Worldwide, intake of added sugars has increased dramatically during the past few decades [4].

In response, the World Health Organisation (WHO) in 2015 issued sugar guidelines, recommending that adults and children restrict their added sugar intake to less than 10% of total energy intake per day, and suggests a further reduction to below 5% [5]. In the United Kingdom (UK), the Scientific Advisory Committee on Nutrition (SACN) recommends that added sugars should account for no more than 5% daily energy intake [3].

Data from the National Diet and Nutrition Survey in the UK show that one of the main sources of added sugars in the diet are sugar-sweetened beverages (SSBs) [6]. In order to achieve a reduction in sugar intake, public health policies promoting SSB reduction are on the increase. Consequently, the food industry is responding in multiple ways, including investing in the formulation of artificially sweetened food products, promoting them as healthier alternatives [4].

Current guidelines developed for public health authorities and consumers consistently recommend a reduction in sugar consumption and recommend artificial sweeteners within foods as a healthy alternative [7]. As a substitute for SSB, LCBs offer the potential to satisfy both thirst and an innate desire for sweetness with minimal caloric load [8,9]; however, their effects on diet quality, weight control and cardiometabolic biomarkers continue to be debated. This study therefore aims to verify the association between LCB consumption, diet quality and cardiometabolic risk factors in British adults.

## 2. Materials and Methods

### 2.1. Study Design

We carried out a data analysis on a cross-sectional study based on two waves (2008–2012 and 2013–2014) of the UK National Diet and Nutrition Survey (NDNS). The NDNS is an annual rolling cross-sectional survey carried out on behalf of Public Health England and the Food Standards Agency. It is designed to assess the diet, nutrient intake and nutritional status of a representative sample of UK adults and children. Households were randomly sampled from the U.K. Postcode Address File, with one adult and one child (18 months or older) or one child selected for inclusion. We included all subjects aged 16 and older at the time of interview.

### 2.2. Interview

Sociodemographic data, lifestyle behaviours, dietary habits, use of medications and dietary supplements were collected during a computer-assisted personal interview.

### 2.3. Dietary Records

Respondents were asked to complete a dietary record for four consecutive days (including weekends and weekdays), giving a detailed description of each item consumed, the time of consumption, and amount, using household measures and photographs. Information on missing food items was collected on repeat visits by interviewers. Trained diet coders then entered the food intake data from completed recordings using an in-house dietary assessment system (Data In, Nutrients Out—DINO). 

From the NDNS archives we retrieved average daily energy intake, protein, total carbohydrate, total sugar, intrinsic sugar, free sugar, total fat, monounsaturated, *n*-6 and *n*-3 polyunsaturated, saturated and trans-fatty acids, fibre, sodium and alcohol intake.

Sugar refers to free or added sugars as defined in the NDNS archives as non-milk extrinsic sugars, comprised either sugars added or naturally present to foods, excluding extrinsic sugars in milk and milk products.

### 2.4. Anthropometric Measurements

Weight, height and waist circumference were taken by trained nurses for those participants who completed 90% of the dietary record. BMI was calculated in kg/m^2^ from weight and height measurements.

### 2.5. Blood Samples

Fasting blood was collected for all participants during the nurse second visit. The following variables were considered in this study: plasma glucose, total cholesterol, low-density lipoproteins (LDL), high-density lipoproteins (HDL) and triglycerides.

### 2.6. Classification of Participants

Subjects were classified into four groups according to beverage consumption over the 4-day dietary record:

(1) LCB group—subjects who consumed LCB (average LCB intake > 0 g/day and average SSB = 0 g/day);
(2) SSB group—subjects who consumed only sugar-sweetened beverage (average LCB intake = 0 g/day and average SSB > 0 g/day);
(3) BB group—subject consuming both types of beverages (average LCB intake > 0 g/day and average SSB > 0 g/day);
(4) NC group—subjects who did not consume either LCB or SSB (average LCB intake = 0 g/day and average SSB = 0 g/day).

LCBs were defined as low- or no-calorie drinks (without added sugar or sugar-free), including carbonated, ready-to-drink and concentrated soft drinks and squashes, excluding water. SSBs were defined as drinks that are not low calorie, with a range of sugar content, carbonated and still, ready to drink and diluted, excluding water. Tea, coffee, fruit and vegetable juices, milk and alcoholic beverages were not considered.

### 2.7. Data Analysis

The response variables considered in this study were: nutrient intake expressed as percentage of total energy intake, UK recommendations for free sugar, saturated fatty acid and fibre intake [10], plasma glucose and lipid profile. Basic characteristics of the population were presented as counts and percentages and compared between LCB and SSB groups by Chi-squared test. To estimate differences in nutrient intake or plasma glucose and lipid profile across beverage consumption groups, we fitted multiple linear regression models. To determine if the differences across groups were statistically significant, we used the Chi-squared test between two nested models (including or not the group variable in the model). We estimated the odds ratio (OR) of being compliant with the UK recommendation for free sugar, saturated fatty acids and fibre intake by using multiple logistic regression models. All models were adjusted for sex, age groups, socioeconomic status and BMI. 

We also carried out stratified analyses for free sugar consumption across strata of sex, age group (16–24, 25–49, 50–64, ≥65 years), BMI category [normal weight (BMI < 25 kg/m^2^), overweight (BMI ≥ 25 and <30 kg/m^2^) and obese (BMI ≥ 30 kg/m^2^)] and socioeconomic status. Socioeconomic status was based on the following categories of the National Statistics socioeconomic classification: (1) Higher managerial and professional occupations, (2) Lower managerial and professional occupations, (3) Intermediate occupations, (4) Small employers and own account workers, (5) Lower supervisory and technical occupations, (6) Semi-routine occupations, (7) Routine occupations. To test the heterogeneity of the group effect in each stratifying variable, we used a Chi-squared test, comparing two nested models, one including the interaction between the beverage consumption group and the stratifying variable and the other not including the interaction term. All statistical tests were two-sided and *p* values < 0.05 were considered statistically significant. The analysis was performed using R version 3.5.0.

## 3. Results

### 3.1. Study Population

We included 5521 subjects who completed the four-day dietary record, of whom 17.0% were classified in the LCB group, 29% in the SSB group, 19.8% in the BB group and 34.2% in the NC group. The median (interquartile range) intake of LCB was 207 mL/day (100–426) in the LCB group, 198 mL/day (83–398) in the BB group. The median (interquartile range) intake of SSB was 169 mL/day (83–373) in the SSB group, 163 mL/day (83–330) in the BB group.

Table 1 gives their sociodemographic characteristics, BMI and smoking status according to group of beverage consumption. Compared with the SSB and NC group, subjects consuming LCB were more likely to be women, in the age category 25–49 years, white and obese, while there were no significant differences in terms of socioeconomic status.

### 3.2. Nutrients Intake

Table 2 shows the average nutrient intake computed over a four-day dietary record across beverage consumption groups. Compared to the SSB group, subjects in the LCB group had a lower energy intake (−173 kcal, IQR: −212; −133), as well as a lower intake of total carbohydrates (−1.7% of energy intake, IQR: −2.3; −1.1), sugar intake (−4.4% of energy intake, IQR: −5.0; −3.9), intrinsic sugar intake (−1.1% of energy intake, IQR: 0.8; 1.4), free sugar intake (−5.6% of energy intake, IQR: −6.1; −5.1) and alcohol (−1.6 g, IQR: −3.2; 0), while protein (+1.70% of energy intake, IQR: 1.4; 2.0) and fibre intakes (+0.4 g, IQR: 0; 0.8) were slightly higher. Compared to the NC group, the LCB group had a slightly increased sodium intake. Conversely, intakes of other nutrients were not substantially different across groups.

**Figure 1:**
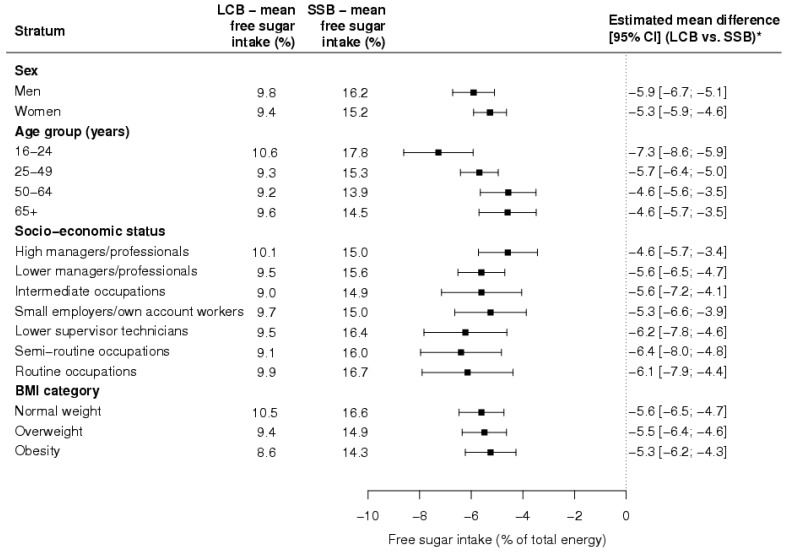
Differences in free sugar intake between the low-calorie (LCB) and sugar-sweetened beverage (SSB) group and corresponding 95% confidence intervals (CIs) according to strata of sex, age, socioeconomic status and body mass index (BMI). * The estimates were obtained by multiple linear regression models adjusted for age, socioeconomic status and BMI (for sex strata); adjusted for sex, socioeconomic status and BMI (for age strata); adjusted for sex, age, and BMI (for socioeconomic status strata); adjusted for sex, age, and socioeconomic status (for BMI strata).

The difference in free sugar intake between the LCB and SSB group was similar across strata of sex (*p* for the interaction = 0.300), socioeconomic status (*p* for the interaction = 0.140) and BMI category (*p* for the interaction = 0.630), whereas it was more pronounced in the young as compared to older individuals (*p* for the interaction = 0.006) (Figure 1).

### 3.3. UK Recommendation for Free Sugar Intake, Saturated Fatty Acids and Fibre

Table 3 gives the percentages of subjects not exceeding the UK recommendation for free sugar and saturated fatty acid intake, and meeting the minimum recommended level of fibre intake. The percentage of people meeting the UK recommendation for free sugar was very low in all groups, although the odds of meeting the UK recommendation were remarkably higher in the LCB as compared to the SSB group (adjusted OR: 9.4, 95% CI: 6.5–13.6). The percentages of subjects within the UK recommendation for saturated fatty acid intake were similar across groups. Only a few people were within the UK recommendation for fibre intake, with no differences across groups. There were no significant differences between LCB and NC group in the percentage people meeting the UK recommendation for free sugars, saturated fatty acids and fibre.

### 3.4. Plasma Glucose and Lipid Profile

Table 4 shows fasting plasma glucose and lipid profile according to beverage consumption groups. The were no significant differences in plasma glucose, total cholesterol, LDL, HDL and triglycerides levels among groups.

## 4. Discussion

This study examined the association between LCB consumption, diet quality and cardiometabolic risk factors in British adults. It found that compared to the SSB group, subjects in the LCB group had a lower energy intake as well as a diet lower in total sugar and free sugars, with an increased odds of meeting current UK dietary guidelines on free sugar intake. Moreover, there were no differences in blood glucose, triglycerides, total cholesterol, LDL, or HDL levels between LCB and SSB or NC group. 

A limited number of studies have examined the associations of SSB/LCB consumption with diet quality and cardiometabolic indicators [11,12,13]. Our findings are in line with studies supporting the hypothesis that replacing SSB with LCB leads to a reduced energy intake and an improved dietary quality in adults. Evidence from the Choose Healthy Options Consciously Everyday (CHOICE) randomised control trial indicated that those who replaced SSBs with either LCB or water also reduced their consumption of added sugar and desserts with the LCB group sustaining a larger reduction in desserts than the water group [14]. Data from the National Health and Nutrition Examination Survey (1999–2008 NHANES, *n* = 22,231) showed that LCB consumers had better Healthy Eating Index subscores for vegetables, whole grains and low-fat dairy, whereas they had a higher intake of saturated fatty acids and sodium [15]. Conversely, we did not find that LCB consumers had a higher intake of saturated fat and sodium. A recent study using the UK National Dietary and Nutrition Survey (2008–2011) also showed that in all main respects (energy, macronutrient and micronutrient intakes) the diets of the LCB group were similar to those who consumed no soft drinks at all (NC). It also showed that LCB consumers did not compensate for the sugar and energy deficit [8].

Our findings are also similar to those from a recent systematic review and meta-analysis [9], based on 129 short-term randomised controlled trials in children and adults, reported that the consumption of low-calorie sweeteners in place of sugar reduces energy intake and body weight. The meta-analysis reported that the consumption of low-energy sweeteners versus sugar-sweetened food before an ad libitum meal reduced energy intake by 94 kcal (95% CI −122 to −66), with no difference versus water (−2 kcal, 95% CI −30 to 26). The meta-analysis of nine sustained intervention randomised controlled trials (4 weeks to 40 months) showed that consumption of low energy sweeteners versus sugar led to a reduction in body weight (−1.35 kg, 95% CI −2.28 to −0.42) that was comparable to that observed when sugar was replaced with water (−1.24 kg, 95% CI −2.22 to −0.26).

On the other hand, other studies have reported a positive association between LCB consumption and BMI and weight gain over time questioning the benefit of LCB for weight management in the long term [12,16,17,18]. It is also postulated there may be differences in cognitive behaviour between subjects in a randomised trial and free-living subjects as to how they use LCB in the context of their diet [19]. Further evidence also suggests that consumption of low-calorie sweeteners may result in complete caloric compensation from other sources [11,20,21]. In addition, findings from a recent study reported that for morbidly obese subjects the use of low-calorie sweeteners was associated with an unhealthy lifestyle and unfavourable eating habits (increased energy intake including sugar and reduced intake of some vitamins) [22]. However, our findings indicate that consumption of low-calorie sweeteners did not result in poorer dietary quality for these subjects.

There is limited and inconsistent research examining the health impact (in particular, related to cardiometabolic indicators) of low-calorie sweeteners. In addition, many of them have focused on children or adolescents and not adults [12]. Positive associations between the use of artificial sweeteners and glucose tolerance [23,24] and hypertension [25] have been identified from observational studies and clinical trials [23,26,27]. A recent child-focused study using NDNS data (2008–2012) had contrasting findings to our study. It reported that SSB intake is associated with higher sugar intake. However, in that study both SSB and LCB intake were linked with less healthy cardiometabolic profiles [24].

In contrast, a recent review [28] including 372 studies (15 systematic reviews, 155 randomised controlled trials, 23 non-randomised controlled trials, 57 cohort studies, 52 case-control studies, 28 cross sectional studies, 42 case series/case reports) found that in healthy subjects, there was no conclusive evidence for the harmful effects of low-calorie sweeteners. In subjects with diabetes and hypertension, the evidence regarding the health outcomes of low-calorie sweeteners was also found to be inconsistent. This review also highlighted the large heterogeneity in studies that could be related to different studied populations, age-related differences in dietary patterns, frequency of low-calorie sweetener use, the need to examine cardiometabolic effects in the context of broader health behaviours, and publication bias [12].

## 5. Strengths and Limitations

The study has important strengths. First, analyses were based on the NDNS data, a high quality nationally representative UK data source. Results are thus generalizable on a population level and can be compared to other recent studies. Second, food and nutrient data were gathered from a self-reported four-day diary, which provides better representation of usual consumption than food frequency questionnaries or 24-h dietary recalls, commonly used in epidemiological studies. However, it is known that food diary may also be somewhat inaccurate in estimating food and nutrient consumption, including sugars. In addition, the increasing use of a mixture of sugars and low-calorie sweeteners within many beverages may have added complexity to the analysis. For example, fruit juices, which were not considered in our analysis, could be an important source of added sugars and, increasingly, low-calorie sweeteners.

Given the cross-sectional nature of the NDNS survey, we cannot rule out reverse causality for some of the study outcomes, such as obesity and other cardiometabolic indices [12,28]. However, randomised trials within this area are also limited by the short or medium-term evaluation of interventions [19].

Finally, we did not consider the contribution of physical activity that may also have affected cardiometabolic risk factors. 

## 6. Conclusions

This study adds to the body of evidence that LCB can have a positive impact on diet quality. Future studies need to be rigorous in design, including well-defined interventions (i.e., providing information of type and dosage of low-calorie sweeteners in the whole diet) and controls. Research should also investigate the long-term effects of using low-calorie sweeteners on specific population groups having multiple comorbidities, including diabetes and metabolic syndrome.

## Figures and Tables

**Table 1:** Characteristics of the population according to type of beverage consumption.

	Number of Subjects (*N* = 5521)				% of the Population				*p* Value ^a^
	BB	LCB	NC	SSB	BB	LCB	NC	SSB	
Number of subjects	1095	936	1887	1603	100	100	100	100	
Sex									<0.0001
Men	449	326	819	739	41.0	34.8	43.4	46.1	
Women	646	610	1068	864	59.0	65.2	56.6	53.9	
Age category (years)									<0.0001
16–24	399	137	146	441	36.4	14.6	7.7	27.5	
25–49	482	454	579	634	44.0	48.5	30.7	39.6	
50–64	131	208	556	278	12.0	22.2	29.5	17.3	
65–96	83	137	606	250	7.6	14.6	32.1	15.6	
Race									<0.0001
White	1035	894	1752	1454	94.5	95.5	92.8	90.7	
Mixed ethnic group	17	10	11	20	1.6	1.1	0.6	1.2	
Black or Black British	13	10	39	47	1.2	1.1	2.1	2.9	
Asian or Asian British	22	13	51	55	2.0	1.4	2.7	3.4	
Any other group	8	9	34	27	0.7	1.0	1.8	1.7	
SES									0.08
Higher managerial and professional occupations	133	162	269	229	12.1	17.3	14.3	14.3	
Lower managerial and professional occupations	299	236	433	399	27.3	25.2	22.9	24.9	
Intermediate occupations	113	98	171	166	10.3	10.5	9.1	10.4	
Small employers and own account workers	121	98	211	155	11.1	10.5	11.2	9.7	
Lower supervisory and technical occupations	112	95	181	154	10.2	10.1	9.6	9.6	
Semi-routine occupations	145	118	275	224	13.2	12.6	14.6	14.0	
Routine occupations	126	96	245	187	11.5	10.3	13.0	11.7	
Never worked	23	18	62	44	2.1	1.9	3.3	2.7	
Other	23	12	34	42	2.1	1.3	1.8	2.6	
Not answer	0	1	2	1	0.0	0.1	0.1	0.1	
Not available	0	2	4	2	0.0	0.2	0.2	0.1	
BMI category									<0.0001
Normal weight (BMI < 25 kg/m^2^)	430	273	605	679	39.3	29.2	32.1	42.4	
Overweight (BMI: 25–29 kg/m^2^)	305	319	633	459	27.9	34.1	33.5	28.6	
Obese (BMI ≥ 30 kg/m^2^)	283	270	478	323	25.8	28.8	25.3	20.1	
Missing	77	74	171	142	7.0	7.9	9.1	8.9	

^a^*χ*^2^ test.

**Table 2:** Energy and nutrients intake according to type of beverage consumption.

	BB	LCB	NC	SSB	Across Group Difference *p* value ^a^	Estimated Adjusted Difference between LCB and SSB (95% CI) ^b^	Estimated Adjusted Difference between LCB and NC (95% CI) ^b^
Energy (Kcal)	1903 (564)	1651 (514)	1673 (514)	1872 (593)	<0.0001	−173 (−212; −133)	+2 (−37; 42)
Carbohydrates (% of energy)	50.7 (7.3)	48.7 (7.8)	48.3 (8.2)	50.7 (7.7)	<0.0001	−1.7 (−2.3; −1.1)	0 (−0.6; 0.6)
Sugars (% of energy)	22.1 (6.6)	18.7 (6.5)	19.7 (6.8)	23.3 (7.2)	<0.0001	−4.4 (−5.0; −3.9)	−0.8 (−1.3; −0.2)
Intrinsic sugars (% of energy)	7.2 (3.7)	9.2 (4.4)	9.7 (4.7)	7.6 (3.9)	<0.0001	−1.1 (0.8; 1.4)	−0.2 (−0.5; 0.1)
Free sugars (% of energy)	14.9 (6.5)	9.5 (5.1)	10.1 (5.7)	15.6 (7)	<0.0001	−5.6 (−6.1; −5.1)	−0.6 (−1.1; −0.1)
Proteins (% of energy)	15.6 (3.4)	17.5 (4.2)	17 (4)	15.5 (3.3)	<0.0001	+1.70 (1.4; 2.0)	+0.4 (0.1; 0.7)
Fats (% of energy)	33 (5.7)	33.1 (6.6)	33.6 (6.6)	33.2 (6)	0.25	0 (−0.5; 0.6)	−0.3 (−0.8; 0.2)
Monounsaturated fatty acids (% of energy)	12.3 (2.5)	11.9 (2.8)	11.9 (2.8)	12.1 (2.7)	0.74	−0.1 (−0.3; 0.1)	−0.1 (−0.3; 0.1)
Polyunsaturated n-6 fatty acids (% of energy)	4.7 (1.3)	4.7 (1.4)	4.7 (1.6)	4.7 (1.5)	0.049	+0.1 (−0.1; 0.2)	−0.1 (−0.2; 0.1)
Polyunsaturated n-3 fatty acids (% of energy)	0.9 (0.4)	1.0 (0.4)	1.0 (0.5)	0.9 (0.4)	0.0003	+0.03 (0; 0.07)	−0.02 (−0.05; 0.01)
Saturated fatty acids (% of energy)	12.1 (3)	12.3 (3.4)	12.7 (3.6)	12.4 (3.2)	0.66	0 (−0.2; 0.3)	−0.1 (−0.3; 0.2)
Trans-fatty acids (% of energy)	0.6 (0.3)	0.6 (0.3)	0.6 (0.3)	0.6 (0.3)	0.76	−0.01 (−0.03; 0.01)	0 (−0.03; 0.02)
Fibre (g)	13.1 (4.6)	13.3 (4.9)	13.6 (5.2)	12.9 (4.9)	0.003	+0.4 (0; 0.8)	0 (−0.4; 0.3)
Sodium (mg)	2.4 (0.8)	2.1 (0.8)	2.0 (0.8)	2.2 (0.8)	<0.0001	-66 (−126; −6)	+109 (49; 168)
Alcohol (g)	11.4 (19.9)	9.6 (18.5)	11.2 (21.1)	11.2 (21.7)	0.003	−1.6 (−3.2; 0)	−0.7 (−2.3; 0.9)

**^a^** Group differences were tested using analysis of covariance; **^b^** between group differences were estimated by multiple linear regression models adjusted for sex, five-year age category, socioeconomic status and BMI category.

**Table 3:** UK recommendation on free sugars, saturated fatty acids and fibre intake according to type of beverage consumption.

		BB	LCB	NC	SSB	Adjusted OR (95% CI) (LCB vs. SSB) ^a^	Adjusted OR (95% CI) (LCB vs. NC) ^a^
Free sugar	Within the UK recommendation	27 (2.5)	180 (19.2)	359 (19.0)	36 (2.2)	9.39 (6.47–13.63)	0.94 (0.76–1.16)
	Over the UK recommendation	1068 (97.5)	756 (80.8)	1528 (81.0)	1567 (97.8)		
Saturated fatty acids	Within the UK recommendation	270 (24.7)	235 (25.1)	431 (22.8)	363 (22.6)	1.10 (0.90–1.33)	1.01 (0.83–1.21)
	Over the UK recommendation	825 (75.3)	701 (74.9)	1456 (77.2)	1240 (77.4)		
Fibre	Within the UK recommendation	2 (0.2)	6 (0.6)	8 (0.4)	12 (0.7)	0.90 (0.33–2.48)	1.76 (0.59–5.27)
	Below the UK recommendation	1093 (99.8)	930 (99.4)	1879 (99.6)	1591 (99.3)		

**^a^** ORs were estimated by multiple linear regression models adjusted for sex, five-year age category, socioeconomic status and BMI category.

**Table 4:** Plasma glucose and lipid profile according to type of beverage consumption.

	BB	LCB	NC	SSB	Across Group Difference *p* value ^a^	Estimated Adjusted Difference between LCB and SSB (95% CI) ^b^	Estimated Adjusted Difference between LCB and NC (95% CI) ^b^
Plasma glucose (mmol/L)	5.07 (0.73)	5.34 (1.22)	5.36 (1.28)	5.16 (1.08)	0.30	0.20 (−0.05; 0.46)	0.16 (−0.10; 0.41)
Total cholesterol (mmol/L)	4.84 (1.06)	5.04 (1.11)	5.14 (1.17)	5 (1.13)	0.62	0.07 (−0.17; 0.32)	0.10 (−0.15; 0.34)
LDL (mmol/L)	2.91 (0.9)	3.03 (0.93)	3.11 (1.03)	3 (0.97)	0.64	0.06 (−0.11; 0.22)	0.06 (−0.10; 0.23)
HDL (mmol/L)	1.42 (0.40)	1.43 (0.42)	1.49 (0.46)	1.47 (0.42)	0.76	0.03 (−0.07; 0.13)	0.02 (−0.08; 0.12)
Triglycerides (mmol/L)	1.22 (0.84)	1.35 (0.93)	1.27 (0.82)	1.25 (0.83)	0.17	0.07 (−0.03; 0.171)	0.10 (0; 0.20)

**^a^** Group differences were tested using analysis of covariance; **^b^** between group differences were estimated by multiple linear regression models adjusted for sex, five-year age category, socioeconomic status and BMI category.
